# Delineating the contours of citizen science: Development of the ECSA characteristics of citizen science

**DOI:** 10.12688/openreseurope.19411.3

**Published:** 2026-03-09

**Authors:** Dilek FRAISL, Muki Haklay, Gerid Hager, Uta Wehn, Linda See, Susanne Hecker, Bastian Greshake Tzovaras, Margaret Gold, Luigi Ceccaroni, Barbara Kieslinger, Sasha Woods, Christian Nold, Bálint Balázs, Marzia Mazzonetto, Simone Rüfenacht, Lea A. Shanley, Alice Motion, Andrea Sforzi, Daniel Dörler, Florian Heigl, Katrin Vohland, Katherin Wagenknecht, Teresa Schaefer, Dorte Riemenschneider, Ariel B. Lindner, Maike Weißpflug, Monika Mačiulienė

**Affiliations:** 1 International Institute for Applied Systems Analysis (IIASA), Laxenburg, Austria; 2 Citizen Science Global Partnership (CSGP), Laxenburg, Austria; 3 University College London Department of Geography, London, England, UK; 4 Research Unit Learning Transitions, Learning Planet Institute, Paris, France; 5 IHE Delft Institute for Water Education, Delft, South Holland, The Netherlands; 6 Centre for Science and Technology Studies (CWTS), Leiden University, Leiden, The Netherlands; 7 Museum für Naturkunde, Leibniz Institute for Evolution and Biodiversity Science, Berlin, Germany; 8 Department of Computational, Quantitative and Synthetic Biology, Sorbonne Université,CNRS, ERL U1338 Inserm, Paris, France; 9 Citizen Science Lab, Leiden University, Leiden, The Netherlands; 10 Earthwatch Institute Europe, Oxford, England, UK; 11 Centre for Social Innovation GmbH, Vienna, Austria; 12 The Open University, Milton Keynes, School of Engineering & Innovation, England, UK; 13 Environmental Social Science Research Group, Budapest, Hungary; 14 Stickydot srl, Brussels, Belgium; 15 European Citizen Science Association, Berlin, Germany; 16 International Computer Science Institute, Berkeley, California, USA; 17 The University of Sydney School of Chemistry, Sydney, New South Wales, Australia; 18 Maremma Natural History Museum, Grosseto, Italy; 19 BOKU University, Vienna, Austria; 20 Natural History Museum Vienna, Vienna, Austria; 21 Federal Office for the Safety of Nuclear Waste Management, Berlin, Germany; 22 Kaunas University of Technology, Kaunas, Lithuania

**Keywords:** citizen science, ECSA 10 Principles of Citizen Science, citizen science terms and definitions

## Abstract

**Background:**

Citizen science is increasingly recognized as a valuable scientific approach across disciplines, contexts, and research areas. However, its rapid expansion and diverse methodologies make it challenging to establish a single definition or universal criteria for what constitutes citizen science. Building on our previously published work that detailed findings from a vignette study used to identify the ECSA Characteristics, this paper specifically focuses on the descriptive results from that study and examines the Characteristics in relation to the ECSA 10 Principles of Citizen Science.

**Methods:**

We developed the ECSA Characteristics through a vignette study, a survey method that captures diverse perspectives on complex topics. We then reviewed the ECSA 10 Principles of Citizen Science, a broad framework for best practices in citizen science, to identify its gaps and limitations, showing how the ECSA Characteristics can help address them.

**Results:**

The results highlight the disciplinary distinctions as well as ambiguities surrounding various citizen science practices. In this context, it is beneficial to adopt an inclusive approach and language that allows the audience to define its own criteria depending on its needs, intended use and specific circumstances.

**Conclusions:**

The ECSA Characteristics were developed in a spirit of openness; identifying areas with diverse and even conflicting views was central to this practice. We recommend their use as a whole set and contend that no one area or characteristic is more important than the other. They should be considered as a toolkit with examples that can guide efforts towards defining citizen science for a specific context and purpose. They are built on the ECSA 10 Principles, addressing some of their gaps and limitations, while at the same time acknowledging the need to update and improve the 10 Principles based on developments in the field.

## Plain Language Summary

Citizen science is increasingly recognized as a valuable method in various research fields, but its definition remains challenging due to the diversity of approaches and practices. This paper introduces the “ECSA Characteristics of Citizen Science”, which outline key elements of citizen science projects. It also connects these Characteristics to the ECSA 10 Principles of Citizen Science, which provide guidance on best practices. Additionally, the paper examines how different groups, such as policymakers and research funders, understand citizen science and highlights the differences in practices across disciplines. A strict definition of citizen science may limit creativity and participation, while a broad one could make it difficult to apply in specific contexts. The paper recommends a flexible, inclusive approach that allows different actors to define citizen science according to their own needs and contexts.

## Introduction

The term “citizen science”, coined independently in the mid-1990s by Alan Irwin and Rick Bonney, is inherently ambiguous. Irwin used the term to describe a form of science that includes knowledge produced and held by citizens, which is valued and respected alongside expert knowledge ( Irwin, 1995). Bonney (1996), highlighted the active participation of volunteers and non-experts in data collection as a demonstration of civic service, enabling research that would not be possible without public participation. Subsequently, citizen science has grown and become established as an approach for knowledge production, accelerated by various technological and societal trends, including the rise of the internet, smartphones and low-cost sensors, and growing access to education ( Fraisl *et al.*, 2022; Pateman *et al.*, 2021; Roser & Ortiz-Ospina, 2016). There are many parallel traditions of public involvement in science resulting in the emergence of diverse terms and definitions, such as community science, participatory action research, crowdsourcing, volunteered geographic information and citizen-generated data ( Beck *et al.*, 2024; Conrad & Hilchey, 2011; Eitzel *et al.*, 2017; MacDonald, 2012; Shirk *et al.*, 2012; Sieber & Haklay, 2015). Concurrently, efforts have been directed toward building a single definition or common criteria for all these activities ( Heigl *et al.*, 2019), although there is also recognition that this might be difficult ( Auerbach *et al.*, 2019; Heigl *et al.*, 2019). More recently, Cooper *et al.* (2021) discussed the efforts to “rebrand” citizen science as “community science” in the United States as the term “citizen” can be considered as a barrier to inclusion. The authors suggested that conversations should focus on approaches and practices that foster inclusion, rather than a name change.

At the same time, a growing body of literature emphasizes that citizen science is not a single, unified mode of knowledge production but a heterogeneous set of practices shaped by distinct approaches, motivations, governance structures, and cultural contexts ( Eitzel *et al.*, 2017; Shirk *et al.*, 2012; Beck *et al.*, 2024). These range from large-scale contributory projects to bottom-up, community-led initiatives, where activities may be embedded in place-based, local or traditional knowledge systems ( Burton *et al.*, 2019; Cooper *et al.*, 2021). In such contexts, efforts to formalize or standardize citizen science have been questioned for potentially privileging institutional norms over alternative ways of knowing and organizing knowledge production (Jopling *et al.*, 2024). As a result, many scholars and practitioners argue for framings that prioritize inclusivity and flexibility, enabling citizen science to be supported and recognised in its full diversity rather than evaluated against a single, prescriptive set of criteria ( Hacking *et al.*, 2024; Haklay *et al.*, 2021).

Overall, the wide range of activities, definitions and terminologies reflect the rapid and ongoing evolution of citizen science as a research field. Since the mid-2010s, the number of initiatives, scientific publications, research topics and policy recognition has grown significantly. For example, in 2015, the White House forum “Open Science and Innovation: Of the People, By the People, For the People”, the launch of CitizenScience.gov and the Federal Crowdsourcing and Citizen Science Toolkit drew significant attention and spurred new activity across and beyond the United States ( Congress.gov, 2015; usa.gov, 2025). In the same year, Europe’s Ten Principles of Citizen Science established best practices for the field ( ECSA, 2015). Professional associations, including the European Citizen Science Association (ECSA), the Association for Advancing Participatory Sciences (former Citizen Science Association) in the United States, the Australian Citizen Science Association and other relevant networks elsewhere, have since expanded capacity, convened conferences, and developed guidance to strengthen support structures and legitimacy of the field in all its diverse forms and modalities ( ECSA, 2025; AAPS, 2025; ACSA, 2025). A global initiative has also emerged through the Citizen Science Global Partnership, launched around the 2017 UN Environment Assembly and formalized as a legal entity in 2022, to coordinate citizen science efforts for sustainable development ( CSGP, 2022). Major international organisations have also elevated citizen science in their frameworks. UNESCO’s 2021 Recommendation on Open Science explicitly recognizes citizen and participatory science ( UNESCO, 2021) and various UN programmes have backed global citizen science initiatives ( Fraisl *et al.*, 2023a; Fraisl *et al.*, 2023b; Fraisl *et al.*, 2025). These organisations and initiatives have both raised the profile of citizen science and produced resources, guidance, platforms, and frameworks that contribute to shaping the understanding of the field and offer practical support for its practice and evaluation.

Domain specific journals, such as Citizen Science Theory and Practice ( Ubiquity Press, 2025) and Community Science ( Wiley, 2025), now offer reputable outlets for disseminating scholarly work and methodological advancements. Visibility has further increased through special issues on citizen science and the SDGs ( Fraisl, 2023), ecology ( Hager & Haywood, 2025; Hoggart, 2021) and biodiversity ( Cigliano *et al.* 2021), science and technology studies ( Schrögel & Kolleck, 2018), social science ( Duží *et al.*, 2022), cities ( Brovelli *et al.*, 2025), and Earth Observation ( Fritz & Fonte, 2016; Fritz & Fonte, 2019). In Europe, successive EU “Citizen Observatory” programmes ( Hager *et al.*, 2021a) and newer Horizon Europe efforts like Urban ReLeaf (2025) and CitiObs (2025) take a comprehensive, socio technical approach, linking citizen participation and data with policy uptake. In parallel, global platforms such as Zooniverse, eBird/Merlin, and iNaturalist now pair massive volunteer communities with AI methods, illustrating powerful forms of human machine collaboration ( CornellLab, 2025; iNaturalist, 2025; Zooniverse Team, 2025). Thematic global initiatives, such as Global Mosquito Alert, demonstrate how national and regional efforts can be networked internationally to tackle shared risks ( UNEP, 2017). Taken together, these organizational and global initiatives complement a growing body of scholarly work that has traced the historical development, scope, and evolution of citizen science, providing insights into the diversity of practices, motivations, and governance approaches in the field. These studies examine, for example, the diversity and evolution of ecological and environmental citizen science ( Pocock *et al.*, 2017), the historical development of citizen science with special attention to its legal and governance dimension ( Berti Suman & Alblas, 2023), shifting academic interpretations of the concept ( Hacking *et al.*, 2024), distinct research strands that employ citizen science approaches ( Kullenberg & Kasperowski, 2016), and the trajectory of the field within education ( Wu & Benaglia, 2024). While these developments reflect growing institutional recognition of citizen science, they also highlight an ongoing tension between efforts to provide shared guidance and the need to preserve openness to diverse, context-specific practices and participant-defined objectives.

Given this evolution, the wide range of topics and domains in which citizens engage in knowledge production, as well as the tension between inclusiveness and attempts to institutionalize the field, reaching consensus on a single definition of citizen science remains difficult. Yet, defining a set of common characteristics can help to identify which activities should be considered as citizen science in its broader sense and to provide a framing for assessing the impacts of such activities. Such a set of characteristics can be beneficial, or even necessary, for stakeholders such as the European Commission that would like to support citizen science activities ( European Commission, 2022) but need guidance on identifying them. National citizen science platforms that feature citizen science projects such as the Austrian “Österreich forscht (Austria is researching)”, German “BürgerSchaffenWissen (Citizens create knowledge)” or Swedish “Medborgarforskning (Citizen Science)” also need to decide which projects they catalogue and curate (Bürger schaffen Wissen, 2024; medborgarforskning. se, 2024; Österreich forscht, 2024). Such stakeholders have varying motivations and objectives for defining citizen science in a more context-specific way, for example, to support policies in a specific domain or national level priorities in a defined research area ( OEAD, 2024). Therefore, it is imperative to embrace the plurality of objectives, needs, and views of different stakeholders to identify common characteristics that can help answer the question “is this citizen science?”.

The ECSA Characteristics of Citizen Science (“the ECSA Characteristics”), the focus of this paper, extend the ECSA 10 Principles of Citizen Science (“the ECSA 10 Principles”) ( ECSA, 2015; Robinson *et al.*, 2018) that are best practice guidance rather than a definition and target mainly practitioners than funders. For the purposes of a broader range of stakeholders, such as policymakers, funders, communities, and scientists new to citizen science seeking to apply these principles in practice, the ECSA 10 Principles are considered too vague to provide adequate guidance for judging proposed citizen science activities or funding applications ( Fraisl *et al.*, 2020b). Additionally, they pay insufficient attention to bottom-up practices such as community-driven initiatives underrepresented in academic science ( Parrish, 2022; Wehn, 2021). Hence, there is a need for guidance through an inclusive set of characteristics of citizen science projects and activities that stakeholders can choose from to make decisions, develop criteria for evaluation, or apply in other practical contexts such as impact assessment ( Dörler *et al.*, 2022). The ECSA Characteristics were developed as a tool to assist defining citizen science within specific contexts and to complement the ECSA 10 Principles. The ECSA Characteristics differ from the ECSA 10 Principles in that they are responsive to each unique context rather than representing a complete set of principles or criteria that initiatives must fulfil.

The aim of this paper is to critically reflect on the ECSA Characteristics, exploring how ambiguities in citizen science practice can be helpful in embracing the plurality of citizen science. Building on our previously published work, which detailed the findings from a vignette study that was used to identify these Characteristics ( Haklay *et al.*, 2021), this paper specifically focuses on the descriptive results from that vignette study. Different from the previous publication, in this paper, we also examine the Characteristics in relation to the ECSA 10 Principles of Citizen Science. We present current and potential applications of ECSA Characteristics along with their opportunities and limitations. Finally, we provide examples of contexts in which the ECSA Characteristics can be particularly helpful, demonstrating how they can supplement and address the shortcomings of the ECSA 10 Principles.

## Method

The methodology consists of two main parts. First, we summarize the methodology used in our previously published study ( Haklay *et al.*, 2021) to provide context for the descriptive results presented here, and outline the additional process we followed to derive the ECSA Characteristics from the existing literature, which was not covered in the earlier study. Second, we identified gaps and limitations in the ECSA 10 Principles that could hinder their practical application due to ambiguity. We present a table listing the 10 Principles and critically analyze these limitations and examine how the ECSA Characteristics can address them by providing additional context and clarity, facilitating the effective implementation of both the Principles and Characteristics, and offering more concrete guidance for use across a variety of citizen science projects.

**Figure 1.**
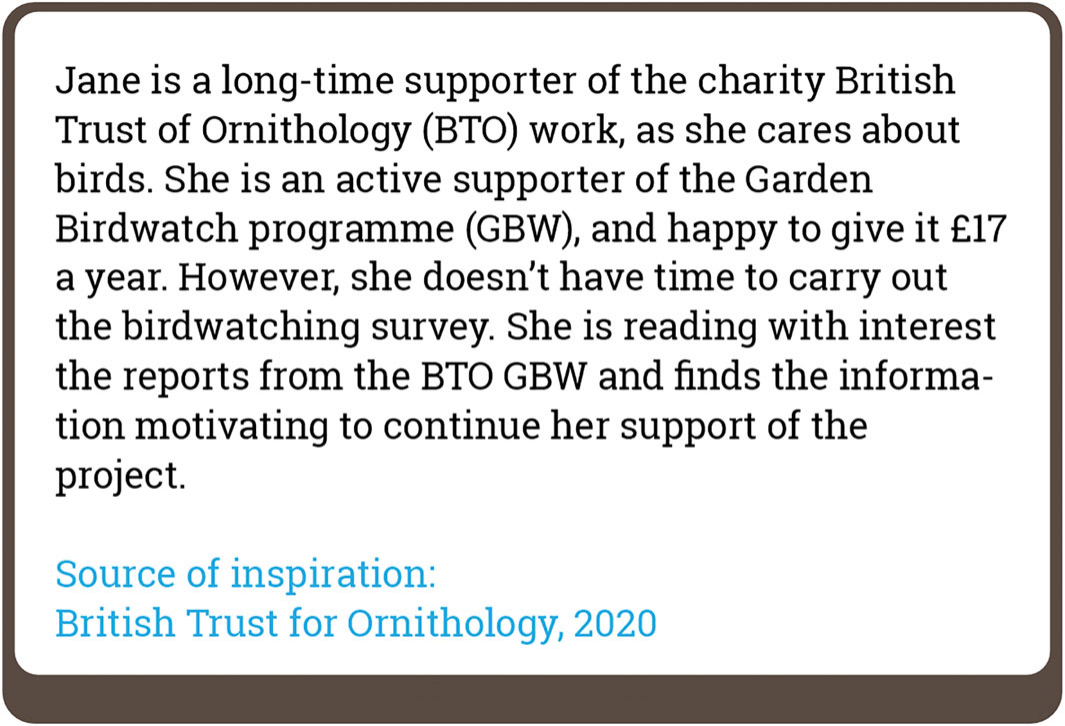
Example of a vignette used in the study (Vignette 3).

We developed the ECSA Characteristics through a vignette study, a form of survey used in healthcare and social studies to allow the extraction of diverse perceptions on complex issues (e.g., Brauer *et al.*, 2009; Taylor, 2006). A vignette study refers to short stories that describe specific circumstances for participants to respond to. Figure 1 is an example of a vignette used in this study. A complete list of 50 vignettes, including clearly recognized citizen science examples, controversial cases where respondents expressed divergent views, and a discussion of how these practices align with or fall outside the common definitions of citizen science, is published in Haklay *et al.* (2020a).

**Figure 2.**
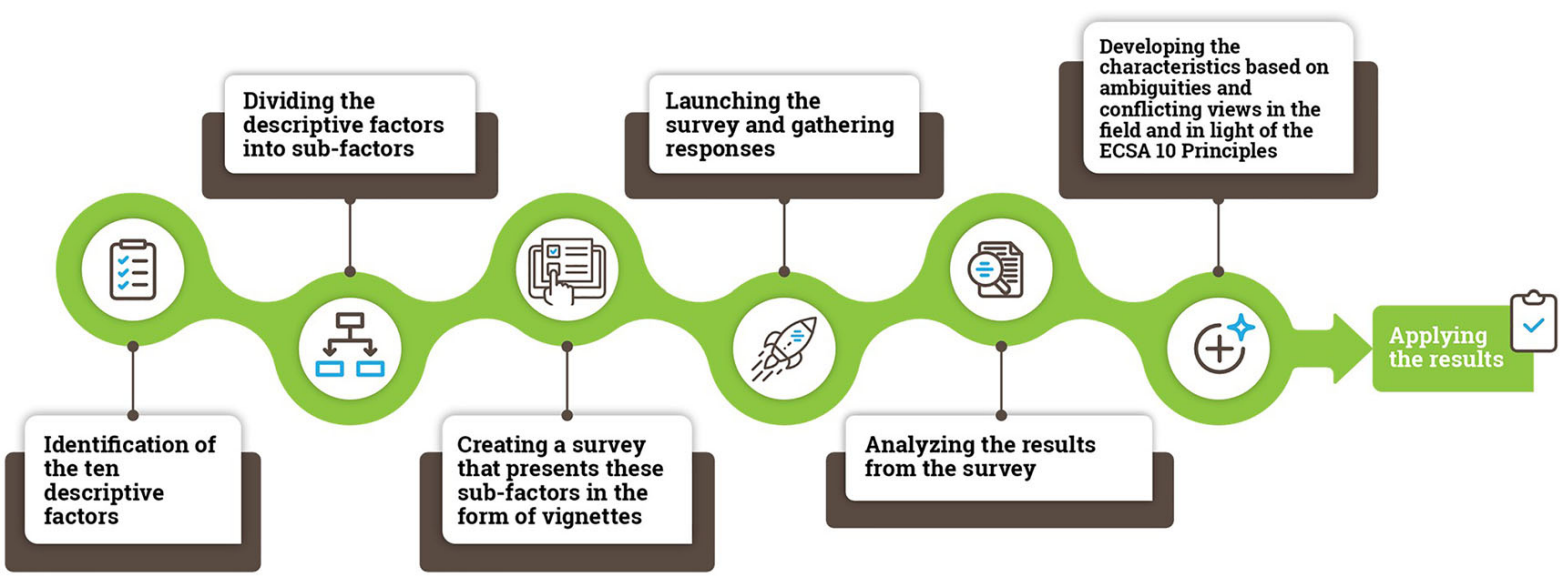
An overview of the methodological process used in the study.

This study should be viewed in the context of the wider methodological process used in this research from the production of vignettes to the development of the ECSA Characteristics ( Figure 2).

The first step included the identification of different dimensions related to citizen science: “descriptive factors”. We identified 10 descriptive factors based on existing typologies in the literature ( Haklay, 2013; Pocock *et al.*, 2017; Shirk *et al.*, 2012; Strasser *et al.*, 2018; Wiggins & Crowston, 2011), and the ongoing debate among citizen science practitioners and researchers ( Auerbach *et al.*, 2019; Heigl *et al.*, 2019) to address controversial issues within citizen science. For example, public participation is a clear requirement for a citizen science project, but uncontroversial so not listed among the ten factors.

In the second step, we divided the ten descriptive factors into 61 sub-factors. A complete set of factors, sub-factors and vignettes used in the study are presented in Supplementary Material 1.

The sub-factors covered issues over which there is some debate in literature and collective experience in the field. For example, the hypothesis-driven experimental (rather than observational) nature of “the Great Grow Experiment” ( Burton *et al.*, 2019) for regenerative growing practices is generally accepted to be citizen science. However, projects where participants receive a (micro) payment for data collection ( Reddy *et al.*, 2010) or where data are harvested from social media are widely debated.

The third step involved the creation of a survey presenting the sub-factors in the form of vignettes. In total, we created 50 vignettes (see Figure 1 for an example). The use of 50 vignettes is the result of the co-occurrence of several of the 61 sub-factors in single vignettes rather than each vignette representing a single sub-factor. In the survey, respondents were asked to indicate the degree to which a vignette can be classified as a citizen science activity on a scale from 0 to 100 ( Liu & Conrad, 2016). Respondents were also requested to rate their level of confidence for their answer for each vignette with the option to explain their decisions.

The fourth step involved survey launch (12 December 2019) on SurveyMonkey. Respondents were recruited through an open social media call (Twitter, LinkedIn and Facebook) and emails to the citizen science mailing lists. More than 330 responses resulted in over 5100 vignette ratings, corresponding to between 90–110 responses per vignette.

In step 5, we analyzed the data, agreed on the structure of the ECSA Characteristics document and discussed respondent comments for each vignette.

Finally, in step 6, we turned results from the analysis into the ECSA Characteristics with consideration of the ECSA 10 Principles. This involved formulating 50% and higher agreement among respondents for each vignette found to be a case of citizen science, and less than 50% agreement for cases found not to represent citizen science, drawing attention to the ambiguities in each case. For example, the degree to which Vignette 3 ( Figure 1) was considered as citizen science was 8.90%. We formulated the ECSA Characteristic related to this, using explanations received from respondents as a basis:

*Financial support for scientific research. Pure financial support to a project, such as crowdfunding, subscription fees and donations, is not considered citizen science, as no participation in any phase of the scientific research takes place. Careful consideration of the consistency with citizen science should be made if the financial contribution is a prerequisite to a form of participation in the scientific research phase of the project.*

To develop a comprehensive set of Characteristics based on the ambiguities and conflicting views from the field, an inclusive process for design and implementation of survey and result production was instigated. Anyone who expressed interest was invited to participate in the study and at any point during the process. Additional efforts to reach out to diverse disciplines and fields where citizen science is prominent were made through networks of the co-authors. This ensured a wide range of perspectives from those using and developing citizen science were considered. Inclusivity was fostered through an international open call to the citizen science and science communication communities. Additional measures to ensure inclusiveness included active consideration of diverse audiences outside the citizen science practitioner and researcher community, such as policymakers and funders, and their potential needs in terms of applying citizen science in their field of work. This was crucial to ensure that established research organizations, Civil Society Organizations (CSOs) and other actors involved in starting and implementing citizen science were represented while creating the ECSA Characteristics. Inclusivity was also ensured while preparing vignettes. The vignettes featured examples of diverse citizen science practices and close attention was paid to the use of inclusive terminology throughout, to reduce the perception that words like “science” or “research” excluded any stakeholder groups, disciplines, fields or initiatives.

We developed the ECSA Characteristics with future applications in mind. Growth in the field of citizen science is opening up new areas, activities, and interactions between people, science, and technology, such as Artificial Intelligence ( Franzen *et al.*, 2021; Ponti & Seredko, 2022), to which the ECSA Characteristics should be applicable. The ECSA Characteristics should also be applicable in a modular way; responsive to unique contexts rather than a set of criteria that initiatives must fulfil to be considered citizen science.

We produced the ECSA characteristics as two separate documents. The first document, *ECSA’s Characteristics of Citizen Science*, is succinct so that it is practical and easy to follow by a wide range of audiences, including policymakers, funders, researchers, and the general public. The second document, *ECSA’s Characteristics of Citizen Science: Explanation Notes,* provides context for the production of the first document and describes the link between the ECSA 10 Principles and the ECSA Characteristics. We also included information and guidance on how the ECSA Characteristics can be interpreted and used, as each characteristic constitutes a topic in its own right.

The first full draft of the ECSA Characteristics was shared through an open call to gather feedback and broader engagement and discussion, both regarding the characteristics, and the process of their identification. The document remained open for feedback for around three weeks (February 2020). We then incorporated received feedback and finalized the aforementioned ECSA Characteristics documents.

We published the ECSA Characteristics and their explanation notes openly on the Zenodo repository in April 2020 ( Haklay *et al.*, 2020d), followed by several promotion activities including an ECSA Webinar ( Haklay *et al.*, 2020b) and a session at the Austrian Citizen Science Conference ( Hager *et al.*, 2021b). We then published a scientific paper including a detailed description of our methodology for developing the ECSA Characteristics ( Haklay *et al.*, 2021).

Following this process, we identified the gaps and limitations in the ECSA Principles, which could hinder their practical application due to their ambiguity. We also examined how Characteristics can help address these gaps by providing additional context and clarity, facilitating the effective implementation of both the Characteristics and Principles, and offering more concrete guidance for their use in a variety of citizen science projects.

## Results

As mentioned previously, this paper builds upon several key documents:

• A peer-reviewed paper presenting the findings from an analysis using the vignette method to identify the ECSA Characteristics of Citizen Science ( Haklay *et al.*, 2021).
• The ECSA’s Characteristics of Citizen Science, made publicly available immediately after the completion of this work to serve as a resource for the community to identify what constitutes citizen science ( Haklay *et al.*, 2020c).
• The ECSA’s Characteristics of Citizen Science: Explanation Notes ( Haklay *et al.*, 2020d), which provide a more detailed explanation of the aforementioned Characteristics.
• The ECSA 10 Principles of Citizen Science ( ECSA, 2015), created as core principles to guide good practice in citizen science. However, their broad and vague nature makes it challenging to translate them into specific, actionable guidance for different contexts, hindering effective implementation and the development of concrete guidelines.

While the findings of this study are based on these key documents, here, we present a novel contribution to the field by outlining the Characteristics themselves, providing context for the documents referenced in points three and four. Additionally, this study addresses the ambiguities and analyze the gaps within the ECSA Principles concretely and explain how the Characteristics can help address them.

**Figure 3.**
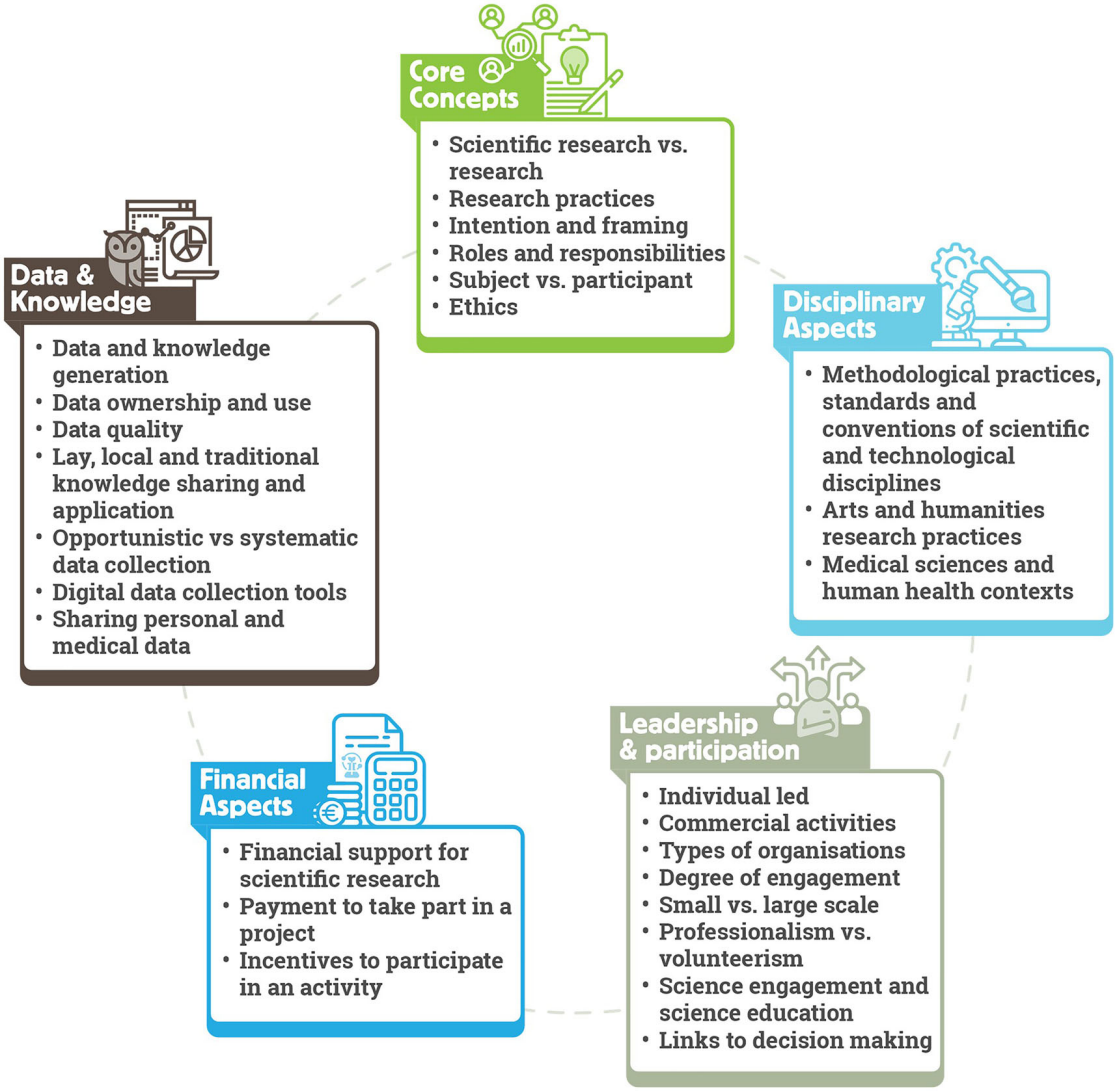
ECSA Characteristics structured in five key areas.

ECSA Characteristics consist of five areas concerning the disagreements regarding what constitutes citizen science: (i) core concepts, (ii) disciplinary aspects, (iii) leadership and participation, (iv) financial aspects, and (v) data and knowledge. Figure 3 provides an overview of Characteristics covered in these areas.

*Core concepts* refer to conceptual issues regarding citizen science. This covers topics including the use of the terms *science* and *research.* The term *science* may result in the perceived exclusion of activities in the fields of humanities, arts, and engineering, as well as grassroots and community-driven initiatives, which can meet all the expectations of scientific practices. We, therefore, use the term *scientific research as* inclusive of all domains of activity, and highlight that citizen science research and practice should follow the protocols and practices of the disciplines to which they belong, to ensure rigor. An additional core concept of citizen science activities is that different actors may be involved in addition to professional scientists and participants, such as community leaders, facilitators, authorities, and other stakeholders. All actors must be made aware of their roles and responsibilities, which is the ethical responsibility of the project owner. Finally, we note that unpacking the core concepts can be particularly challenging in ambiguous areas such as the difference between *subject* and *participant* in the context of the medical or social sciences. We recommend that researchers identify and clearly communicate their perception and framing of the term *participant* and be transparent regarding practices, as essential to the ethical aspects of the citizen science activity.

*Disciplinary aspects* relate to the varying methodologies, standards and common practices applied to the design and running of citizen science projects. For example, requirements related to data management, including quality assurance and quality control processes, may be different for the natural sciences when compared to community-led practices to improve wellbeing. Additionally, some areas of research can be particularly prone to ambiguity. For example, in the arts and humanities, approaches can be more interpretive than descriptive. Therefore, it is important to pay attention to the body of best practice relevant to those research fields and seek to implement them in citizen science projects referring to those domains. We also highlight varying approaches to personal information sharing in citizen science practices. Collection of personal data may be a prerequisite of some activities, such as in the health domain, but may be avoided in projects exploring highly sensitive issues such as gender-based violence. In all cases, it is essential that activities comply with data privacy rules and regulations in the country or countries in which the project operates, and ensure participant safety, security and wellbeing.

The degree of participant engagement from passive to active, the scale of the project from small to large, and the type of organisation leading the project such as grassroot organisations, NGOs, public authorities or academic institutions, are all examples of topics covered under the *leadership and participation* category. Here, we highlight special concerns regarding commercial organisations in citizen science. Examples include data collection through sensors, health monitoring devices and mobile applications. In such cases, it is important to understand how data are used and shared before considering the activity as *citizen science* or *not citizen science.* We recommend applying the ECSA 10 Principles as generic guidance and ascertaining transparency of a project to better understand whether such activities align with the practice of citizen science. For the degree of engagement, active participation that requires the cognitive attention of participants is favored over limited participation such as in volunteered computing projects, where the participation is focused on the participant downloading software to donate their computing power. However, such projects can also be considered citizen science depending on whether the participants are well informed regarding the purpose and process of the research undertaken and how their participation will contribute to that purpose and process.

*Financial aspects* of the ECSA Characteristics include aspects such as donations or payments to participate in a project (for example through membership fees) and conversely payments to participants as incentives. Purely financial contributions from a ‘participant’ to a project through donations or subscription fees without further engagement is not considered citizen science. However, this needs to be evaluated on a case-by-case basis, as projects may still align with the ECSA 10 Principles and the ECSA Characteristics in other ways – for example, if a project requires payment for participation such as purchasing equipment from the project owner to enable data collection. Another ambiguous case widely discussed among the citizen science community is that of financial incentives for participants. In such cases, the local context of the project should be considered, as well as the nature of the task and the contribution of the participants. For example, in some projects, compensation of participants for their time and efforts is appropriate due to their economic situation. In such cases, financial payments need not alter project objectives or convert participants into professional project staff members.

*Data and knowledge* refer to themes from data quality and ownership to sharing of personal and medical data, and how data and knowledge generation issues influence an activity. Here, we emphasize that within citizen science, various forms of data and knowledge generation exist, and that disciplinary contexts and standards must be evaluated, including the data quality requirements. For data ownership and use, citizen science is considered closely linked to open science, including open data sharing and full transparency in ownership. However, sharing of sensitive information and data, such as indigenous data or Traditional Ecological Knowledge (TEK) ( Shanley, 2015), the location of endangered species or personal health measurements in medical health research require special considerations ( Carroll *et al.*, 2020; Fraisl *et al.*, 2022; UNESCO, 2021). Here, we also consider a range of different methods used for data collection in citizen science activities. In some projects, participants are required to use standard protocols to share observations, while in others they are asked to report data opportunistically at random sites and time intervals or self-report on their personal circumstances or experiences. All these approaches can qualify as citizen science depending on the aims and the context of the project, as well as the fitness for purpose of the data.

### The ECSA Characteristics in relation to the ECSA 10 Principles of Citizen Science

The ECSA 10 Principles provide a foundational framework for citizen science, outlining best practices across key areas, such as participant engagement and data management. The ECSA Characteristics complement these Principles, addressing some of their gaps, which arise from their inherent ambiguity and broadness. For example, the first principle emphasizes the importance of actively involving citizens in scientific endeavors. However, the definition of “active involvement” remains unclear. Does simply downloading an app to contribute spare computing power to a research project constitute active involvement or is more hands-on participation necessary, such as submitting plant observations through a mobile app? The ECSA Characteristics highlight the varying degrees of involvement in citizen science, ranging from equal partnership between participants, scientists and other stakeholders to cases where participant contributions mainly involve gathering data or providing computing resources. The Characteristics emphasize the importance of participant awareness regarding their contribution, including in cases where the data they produced is used indirectly or for secondary purposes, such as reusing images shared on social media. Table 1 provides a full list of ECSA 10 Principles, outlining their identified gaps as exemplified by the ambiguity of “active involvement”, and demonstrating how the ECSA Characteristics can help to address these gaps.

**Table 1.** The ECSA 10 Principles, their gaps and how the ECSA Characteristics can help address these gaps.

Principles	Gaps	Characteristics
**1.** Citizen science projects actively involve citizens in scientific endeavour that generates new knowledge or understanding. Citizens may act as contributors, collaborators, or as project leader and have a meaningful role in the project.	• No clarity on what “active” involvement means; • Overemphasis on the “scientific” aspect of citizen science; • Not clear what “new knowledge or understanding” means; • The role of citizens does not reflect the diversity in the field.	• Varying degrees of engagement are highlighted with concrete examples and specific recommendations (Leadership and Participation); • The use of the term “science” is clarified drawing attention to the risk of excluding some citizen science activities (Core Concepts). • Different forms of knowledge generation are described (Data and Knowledge); • The potential roles of citizens, scientists and other actors are covered (Core Concepts).
**2.** Citizen science projects have a genuine science outcome. For example, answering a research question or informing conservation action, management decisions or environmental policy.	• Not clear what “genuine scientific outcome” means; • Overemphasis on “science”; • Outcome examples do not reflect the potential diverse outcomes.	• “Genuine scientific outcome” is clarified (Core Concepts); • How to interpret “science” is explained (Core Concepts); • Various potential outcomes and objectives of citizen science projects are clearly laid out (Core Concepts & Leadership and Participation).
**3.** Both the professional scientists and the citizen scientists benefit from taking part. Benefits may include the publication of research outputs, learning opportunities, personal enjoyment, social benefits, satisfaction through contributing to scientific evidence, e.g., to address local, national and international issues, and through that, the potential to influence policy.	• “Benefit” does not capture the contentious nature incentives for example, although it is a very debated topic in citizen science; • Does not include the diverse set of actors that are/can be involved in a citizen science activity.	• Incentives are covered (Financial Aspects); • Multiple actors that can be involved in citizen science and their roles and responsibilities are discussed (Core Concepts).
**4.** Citizen scientists may, if they wish, participate in multiple stages of the scientific process. This may include developing the research question, designing the method, gathering and analysing data, and communicating the results.	• Implications of participation in one or multiple stages of the project may create confusion such as when the engagement is limited to downloading a software for data collection.	• Participation aspects are discussed, including the conditions in which minimal engagement occurs, and relevant recommendations are provided (Leadership and Participation).
**5.** Citizen scientists receive feedback from the project. For example, how their data are being used and what the research, policy or societal outcomes are.	• Providing feedback to the participants may have ethical and legal implications related to data ownership, sharing and use.	• The issues related to feedback and ethical and legal implications are discussed and the link to the importance of data ownership and use is made (Data and Knowledge).
**6.** Citizen science is considered a research approach like any other, with limitations and biases that should be considered and controlled for. However, unlike traditional research approaches, citizen science provides opportunity for greater public engagement and democratisation of science.	• The limitations and biases need clarity.	• Potential limitations and biases specific to citizen science are highlighted along with the recommendations on how to address them (Leadership and Participation & Data and Knowledge).
**7.** Citizen science project data and metadata are made publicly available and where possible, results are published in an open access format. Data sharing may occur during or after the project, unless there are security or privacy concerns that prevent this.	• Data sharing practices are not covered in sufficient detail and potential ethical and legal aspects are not clear; • No clarity on what the security and privacy concerns can be.	• Data sharing is discussed in detail both in the context of sensitivity based on disciplines such as medical sciences and open science requirements, as well as their ethical and legal aspects (Core Concepts, Disciplinary Aspects & Data and Knowledge).
**8.** Citizen scientists are acknowledged in project results and publications.	• There is no clarity about the privacy and ethical aspects of such an acknowledgment or what it actually refers to.	• The ECSA Characteristics include a short statement about the ethical aspects of citizen science, and indirectly address this. However, since the participation in publication is not a source of controversy, the ECSA Characteristics do not address publications directly.
**9.** Citizen science programs are evaluated for their scientific output, data quality, participant experience and wider societal or policy impact.	• Overemphasis on “science” and data quality rather than the fitness-for- purpose of data.	• How the term “science” should be interpreted is clarified (Core Concepts) and the data quality and fitness-for- purpose of data are discussed (Data and Knowledge).
**10.** The leaders of citizen science projects take into consideration legal and ethical issues surrounding copyright, intellectual property, data sharing agreements, confidentiality, attribution, and the environmental impact of any activities.	• No clarity on how these legal and ethical issues can be taken into consideration.	• Specific sections on legal and ethical issues related to various aspects of citizen science are presented (Core Concepts & Data and Knowledge).

## Discussion

With the publication of the ECSA Characteristics, we have provided conceptual boundaries and guidance that are aimed at assisting others in developing a domain specific definition. Since their publication, the Characteristics have become a valuable resource for the field of citizen science because they have been made publicly available ( Haklay *et al.*, 2020c). They serve as a crucial complement to the ECSA 10 Principles, providing concrete guidance for best practice in citizen science beyond the broad and generic ECSA 10 Principles. Although the ECSA Characteristics have been openly accessible and widely used in the field, as discussed in this section, the findings presented in this paper are novel. They explain how the statistical results from our previous work by Haklay et al. (2021) have been transformed into explanatory outcomes that can help various stakeholders, such as researchers, research funders, and policymakers, to effectively interpret and make informed decisions regarding what constitutes citizen science and what falls outside the scope of this field.

Our findings show that the emphasis on non-strict definitions and the plurality of citizen science initiatives reflected in the ECSA Characteristics is well aligned with a substantial body of literature that identifies heterogeneity as a defining and productive feature of the field. Previous studies have highlighted that citizen science encompasses a wide range of approaches, traditions, governance models, and participation practices, shaped by local social, cultural, and institutional contexts, and that attempts to impose universal or rigid definitions risk marginalizing valuable forms of practice ( Eitzel *et al.*, 2017; Shirk *et al.*, 2012; Cooper *et al.*, 2021). This literature argues for flexible frameworks that enable comparability and shared learning while preserving contextual adaptability, a perspective that underpins the design of the Characteristics and reinforces their potential relevance beyond European policy and research context.

ECSA Characteristics were initially intended to support policy actors, funders, researchers, and practitioners as a guiding framework for consideration when assessing, funding, designing, and implementing citizen science activities, but in reality, their use and application can be much wider.

In terms of current and past applications, the ECSA Characteristics have been used in the EU-Citizen.Science platform and the Measuring the Impact of Citizen Science (MICS) projects funded by the European Commission ( eu-citizen.science, 2023; Parkinson *et al.*, 2022). They have supported the development and content of the EU-Citizen.Science website by providing basis and guidance for identification of good quality resources and projects related to citizen science featured on the platform. To support this, a quality criteria framework was created to define and share good quality resources in Europe and beyond. The first overarching criterion of this framework is to assess whether a resource submitted by a user, for feature on the platform, is related to citizen science, for which the ECSA Characteristics provide guidance. All resources and projects featured on the EU-Citizen.Science platform go through this quality criteria framework, including the use of the ECSA Characteristics along with the ECSA 10 Principles. The quality framework combines top-down and bottom-up approaches: the top-down component includes minimal overarching criteria, which are required criteria for relevance and standard metadata, and a suggested criterion encouraging engagement with the ECSA 10 Principles, followed by specific and supporting criteria assessing aspects such as access, readability, content, applicability, and format quality. Resources must meet at least 50% of the relevant specific criteria to be included, while supporting criteria provide additional guidance without affecting the rating. The bottom-up component engages the community, allowing them to upload, rate, and comment on resources, ensuring inclusivity and responsiveness to diverse needs. By clarifying ambiguities in the 10 Principles and providing context-sensitive guidance, the ECSA Characteristics enable moderators and stakeholders to evaluate resources effectively while accommodating the diversity of citizen science practices ( Fraisl *et al.*, 2020b).

Within the Measuring Impact in Citizen Science project, funded by the European Commission, the ECSA Characteristics framework has guided the development of measurable indicators for citizen-science impact assessment. This operationalizes the ECSA Characteristics, making them applicable in quantitative studies ( Parkinson *et al.*, 2022, [about.mics.tools], and Table 2).

**Table 2.** An example of the quantification of the ECSA Characteristics as MICS indicators.

ECSA Characteristics of citizen science	MICS indicators
**Data and knowledge generation.** Citizen science, scientific, academic and policy-oriented research can include different forms of data and knowledge generation, including novel data generation, creation of new analyses, or production of new knowledge in written and other forms. The knowledge produced in such projects should aspire to disciplinary standards, such as appropriate data quality and quality assurance, the peer review of project publications and materials, or policy-relevant evidence that is fit for decision-making.	What forms of knowledge does the project as a whole create? New data (quantitative or qualitative) New analyses (including existing approaches applied to new data) New methodologies (e.g., for data collection, participant engagement, education) New concepts or theories None of the above I don't know

Additionally, the 50 vignettes developed in the study and based on a defined set of ten descriptive factors, have proven to be a valuable open educational resource, as a basis to discuss different types of citizen science. They have, for example, been used to engage students to reflect on citizen science as part of the “Citizen Science” course taught at University College London, and by the “Taking Citizen Science to Schools” Research Centre of the Technion/University of Haifa. Therefore, the application of the ECSA Characteristics can contribute to the educational curriculum of the next generation of researchers.

The vignettes were also used on several multiple occasions at conferences to facilitate in depth discussion on the nuanced understandings of citizen science ( Hager & Kieslinger, 2020; Hecker *et al.*, 2021). During one such discursive workshop, it became apparent that robust judgement of whether something should be considered citizen science depends on the completeness and quality of information available across all 10 descriptive factors. The debates also suggested nuanced dependencies across factors, with different perceived weights meaning that citizen science classification could change in relation to different factor combinations ( Hager *et al.*, 2021b). This further underlines the complex interplay of considerations when characterizing citizen science in this way, and why there remains potential for disagreement and confusion.

In terms of potential applications, the ECSA Characteristics can be used in studies where the citizen science landscape is analyzed. For example, in their mapping of citizen science contributions to the UN Sustainable Development Goals (SDGs) Global Indicator Framework, Fraisl *et al.* (2020a) reported on the difficulty of citizen science classification for a given project, highlighting cases where authors had disagreement. Similarly, in a subsequent study, Characteristics served as the foundation for determining whether a project qualifies as citizen science ( Fraisl *et al.*, 2023b). Additionally, exploration of citizen science in distinct disciplines and areas of application, such as health, can help guide the framing of research that requires citizen science classification for projects and initiatives. In such cases, ECSA Characteristics provide guidance, especially due to their special focus on the areas of ambiguity. Moreover, researchers new to the field, who want to implement citizen science as a practice in their research, represent another critical audience. For example, UK Research and Innovation (a national funding body) promoted a call for proposals explicit in the aim of introducing new researchers to citizen science ( Natural Heritage Science Forum, 2023; UKRI, 2019). Another example is the role they have played in facilitating the implementation of the UNESCO Recommendation on Open Science ( UNESCO, 2021). The recommendation outlines the close relationship between the Open Science movement and citizen science, and the role of citizen science in opening up the scientific process ( Wehn *et al.*, 2020; Wehn & Hepburn, 2022).

Another potential application is the use of ECSA Characteristics by national citizen science platforms and networks such as the Austrian, German, and Swedish platforms ( Heigl *et al.*, 2020; Swiss Citizen Science Principles Working Group, 2022). As part of the ECSA Working Group “Citizen Science Networks”, owners of these platforms and others developed a set of criteria for the specific purpose of project selection for their platforms ( Dörler *et al.*, 2022). The ECSA Characteristics can support their activities in refining the criteria on which citizen engagement projects, activities, and resources fall under the category of citizen science, as their work is intended to be a living resource, evolving as the field grows, similar to the ECSA Characteristics.

The ECSA Characteristics can also assist funders and decision makers in identifying citizen science activities for their respective funding and policy decisions. Public and philanthropic funders alike are increasingly aware of the role and potential of citizen science to address societal challenges associated with basic and applied research, and development interventions. Similarly, decision makers in public authorities at different governance levels are frequently approached by, involved in, or initiate citizen science activities themselves, but with a similar lack of background on citizen science. In such cases, the ECSA Characteristics can provide a set of guidelines for these actors to define citizen science based on their needs and context.

Finally, the ECSA Characteristics can assist grassroots, community-led initiatives in deciding to frame their initiatives as citizen science, which can help them rebrand, structure or improve their activities for communication and dissemination, fundraising, advocacy and other purposes.

ECSA Characteristics address many limitations of the ECSA Principles, as presented in the Results section and in Table 1. However, one limitation of the ECSA Characteristics is that, despite the effort, they may still be vague for use by communities that require global definitions and methodologies. For example, the UN Statistics Division (UNSD), other UN and international agencies, and the National Statistical Offices around the world seek to mobilize new sources of data to track progress towards the SDGs. These groups have difficulties in “defining” citizen science and its relevant aspects to unlock its potential for SDG monitoring due to the diversity of terms, methodologies and applications. However, for successful official monitoring, global definitions and globally agreed methodologies for data collection are needed to ensure global-level comparisons. The broad and all-inclusive approaches necessary for the development of the field of citizen science make it challenging to address the needs of these official statistics communities. On the other hand, non-strict definitions can also help these authorities to work with the citizen science initiatives at a local and national level, and to address their context- and locality-specific data gaps and needs, which may not be covered in the global methodologies of the SDGs or international frameworks ( Fraisl *et al.*, 2022; Fraisl *et al.*, 2023a). In such cases, it may be helpful for these communities to work with experts who can support the interpretation and use of the ECSA Characteristics in a given context.

Another limitation is that the ECSA Characteristics, like the ECSA 10 Principles, are inherently Europe-centric, as suggested by their name. Although efforts have been made to ensure that the development process has been inclusive and consider diverse geographies as outlined in the methodology section, the fact that EU-Citizen.Science project, which supported the development of the ECSA Characteristics, was funded by the European Commission and involved primarily European partners, means they may still retain a European focus. However, similar to how the Australian CS Association adapted the ECSA 10 Principles to their local context ( ECSA, 2015), comparable efforts could be made for the Characteristics. Moreover, recent discussions among the co-authors of this paper have considered expanding the ECSA 10 Principles and Characteristics to a global framework that considers the unique local contexts of different regions, with the aim of establishing regularly updated global guidelines for best practice in citizen science. Any such effort would require a genuinely inclusive approach, with strong socio-cultural and geographical representation, particularly from the Global South. This effort could be led by a Community of Practice formed by the Citizen Science Global Partnership ( CSGP, 2022), which leads similar efforts such as the Citizen Science and Open Science Community of Practice ( CSGP, 2024). Such an initiative is crucial given the rapidly evolving nature of the field and the fast-paced evolution of technologies such as Artificial Intelligence that directly influence citizen science project development, implementation and associated ethical considerations ( Fortson *et al.*, 2024; Fraisl *et al.*, 2025).

## Conclusion

The ECSA Characteristics were developed in a spirit of openness; identifying areas with diverse and even conflicting views was central to this practice. We recommend their use as a whole set and contend that no one area or characteristic is more important than the other. They should be considered as a toolkit with examples and that can guide efforts towards defining citizen science for a specific context and purpose. They are built on the ECSA 10 Principles, addressing some of their gaps and limitations, while at the same time acknowledging the need to update and improve the 10 Principles based on developments in the field. Most importantly, the ECSA Characteristics complement the ECSA 10 Principles by providing greater specificity, contextual grounding and practical guidance, supporting their clear interpretation, easier application, and the resolution of their ambiguities.

Citizen science is a rapidly growing field that is transforming research while questioning and opening up established practices and processes of conceptualizing and “doing” science. The dynamism of the field makes it difficult to agree on a universal definition and a single set of criteria. Rather than proposing a fixed definition, this paper contributes a set of Characteristics that acknowledge plurality while offering structured guidance. By providing characteristics that embrace diverse practices and activities, the ECSA Characteristics can support various stakeholders, such as funders, scientists or practitioners that are new to the field, in understanding the richness of citizen science practices, and in adapting criteria to specific contexts.

Our findings recognize common practices within citizen science, including environmental and ecological projects centered on data contribution, as well as established approaches rooted in the social sciences, humanities, and grassroots co-creation of knowledge. The framework we propose is flexible enough to accommodate future developments in citizen science, while remaining grounded in core principles aimed at ensuring the highest ethical standards.

Citizen science is still establishing itself as a valid scientific approach in some quarters. While this validation can be assisted through agreement on the borders of the field, this must be inclusive of diverse methods and practices and acknowledge the richness of the field and the need for its further development. The ECSA Characteristics represent an important step toward the fulfilment of this goal by articulating how the ECSA 10 Principles can be interpreted, extended, and applied across varied citizen science contexts, supporting both coherence and diversity within the field.

## Ethical considerations

The survey adhered to data minimization practices, and the analysis did not include personal details, except where respondents explicitly requested to be associated with their contributions. Involvement in the study was based on written informed consent. All survey respondents mentioned by name have explicitly given their consent to be associated with their comments and to retain their name in the deposited file. All participants featured in the video have given their consent for its release. Data protection registration for the project was provided by the Centre for Research and Interdisciplinarity, Université de Paris.

## Data Availability

Zenodo: ECSA Characteristics of Citizen Science. https://zenodo.org/communities/citscicharacteristics/records?q=&l=list&p=1&s=10&sort=newest This project contains the following extended data:
• Supplementary Material 1. (Supplementary Material – Table of Vignettes (case descriptions). https://doi.org/10.5281/zenodo.4281293
•
**Survey Data Set - Would you call this Citizen Science?** https://doi.org/10.5281/zenodo.4266684
•
**Webinar on the Characteristics of Citizen Science** https://doi.org/10.5281/zenodo.3859969
•
**ECSA’s Characteristics of Citizen Science** ( https://doi.org/10.5281/zenodo.3758668) •
**ECSA’s Characteristics of Citizen Science: Explanation Notes** https://doi.org/10.5281/zenodo.3758555 Data are available under the terms of the Creative Commons Attribution 4.0 International license (CC-BY 4.0) ( https://creativecommons.org/licenses/by/4.0/).
