# Supplementary material for: Delineating the contours of citizen science: Development of the ECSA characteristics of citizen science
Source: Open Res Eur. 2026 Apr 24;5:128. Originally published 2025 May 9. [Version 4] doi: 10.12688/openreseurope.19411.4 (PMC12831952; doi:10.12688/openreseurope.19411.4)
Supplement: Peer review history

# Reviewer report: Reviewer response for version 4

**DOI:** 10.21956/openreseurope.25414.r72985

**Published:** 2026-05-05

**Authors:** Isabelle Bonhoure

**Affiliations:** 1 University of Barcelona, Barcelona, Catalonia, Spain

No further comment to make.

If applicable, is the statistical analysis and its interpretation appropriate?

Not applicable

Is the study design appropriate and is the work technically sound?

Partly

Is the work clearly and accurately presented and does it engage with the current literature?

Yes

Are the conclusions drawn adequately supported by the results?

Yes

Are sufficient details of methods and analysis provided to allow replication by others?

Yes

Are all the source data and materials underlying the results available?

Yes

Reviewer Expertise:

citizen science

I confirm that I have read this submission and believe that I have an appropriate level of expertise to confirm that it is of an acceptable scientific standard.

# Reviewer report: Reviewer response for version 4

**DOI:** 10.21956/openreseurope.25414.r72982

**Published:** 2026-04-27

**Authors:** Petteri Laihonen

**Affiliations:** 1 University of Jyväskylä, Jyväskylä, Finland

Thank you for the response, my concerns have now been fully addressed.

If applicable, is the statistical analysis and its interpretation appropriate?

Not applicable

Is the study design appropriate and is the work technically sound?

Partly

Is the work clearly and accurately presented and does it engage with the current literature?

Partly

Are the conclusions drawn adequately supported by the results?

Yes

Are sufficient details of methods and analysis provided to allow replication by others?

Yes

Are all the source data and materials underlying the results available?

Yes

Reviewer Expertise:

Co-creative Citizen Science in Applied Language Studies, Participatory Research

I confirm that I have read this submission and believe that I have an appropriate level of expertise to confirm that it is of an acceptable scientific standard.

# Reviewer report: Reviewer response for version 3

**DOI:** 10.21956/openreseurope.25044.r70827

**Published:** 2026-03-18

**Authors:** Shane Orchard

**Affiliations:** 1 University of Canterbury, Christchurch, New Zealand

The revised version (v3) now incorporates a number of changes in response to reviewer feedback which have improved the manuscript.

If applicable, is the statistical analysis and its interpretation appropriate?

Not applicable

Is the study design appropriate and is the work technically sound?

Partly

Is the work clearly and accurately presented and does it engage with the current literature?

Partly

Are the conclusions drawn adequately supported by the results?

Yes

Are sufficient details of methods and analysis provided to allow replication by others?

Yes

Are all the source data and materials underlying the results available?

Yes

Reviewer Expertise:

Socio-ecology, Participatory Science

I confirm that I have read this submission and believe that I have an appropriate level of expertise to confirm that it is of an acceptable scientific standard.

# Reviewer report: Reviewer response for version 3

**DOI:** 10.21956/openreseurope.25044.r70828

**Published:** 2026-03-17

**Authors:** Petteri Laihonen

**Affiliations:** 1 University of Jyväskylä, Jyväskylä, Finland

I have read the revised version 3, and I have found that the common concerns with other reviewers have been addressed. However, I still found that some important recent literature on research ethics I suggested have been ignored without a response. 

 I still think that both the 

 ALLEA (2023) The European Code of Conduct for Research Integrity – Revised Edition 2023. Berlin. and the 

 The ROSiE General Guidelines on Responsible Open Science (version 2.0; 2025) should be consulted. (Refer to reference no.1&2)

 The ALLEA (2023) code of conduct has most importantly been revised to include Citizen Science and includes the category "participant" not just "research subject". Furthermore, the ROSIE guidelines address many of the same questions discussed in the article. In general including these two sources could improve the discussions on the ethical dimensions of citizen science in the article. At present the article makes frequent reference to ethics, but fails to specify its definition or interpretation behind such statements as in the conclusions: "The framework we propose is flexible enough to accommodate future developments in citizen

 science, while remaining grounded in core principles aimed at ensuring the highest ethical standards." For the reader it remains unclear what the "highest ethical standards" might be?

If applicable, is the statistical analysis and its interpretation appropriate?

Not applicable

Is the study design appropriate and is the work technically sound?

Partly

Is the work clearly and accurately presented and does it engage with the current literature?

Partly

Are the conclusions drawn adequately supported by the results?

Yes

Are sufficient details of methods and analysis provided to allow replication by others?

Yes

Are all the source data and materials underlying the results available?

Yes

Reviewer Expertise:

Co-creative Citizen Science in Applied Language Studies, Participatory Research

I confirm that I have read this submission and believe that I have an appropriate level of expertise to confirm that it is of an acceptable scientific standard, however I have significant reservations, as outlined above.

## References

[1] : The European Code of Conduct for Research Integrity – Revised Edition 2023. *Berlin, DOI 10.26356/ECOC* .2023;

[2] : The ROSiE General Guidelines on Responsible Open Science (version 2.0). *DOI 10.5281/zenodo.10046519* .2025;

# Response

**Published:** 2026-03-19

**Authors:** Dilek FRAISL

We sincerely thank the reviewer for taking the time to evaluate the manuscript again and for noting that their comments have largely been addressed, with the exception of the inclusion of their recommended literature. We would like to clarify that Version 3 of the manuscript was submitted before we received this reviewer’s initial report. The reason many of the reviewer’s comments were already addressed is that another reviewer raised very similar points, which we incorporated during that revision. As a result, Version 3 did not yet include the specific literature suggested by this reviewer. We have now revised the manuscript accordingly and added the two references strongly recommended by the reviewer. We agree that these additions significantly strengthen the paper by incorporating relevant and recent literature on the topic.

# Reviewer report: Reviewer response for version 2

**DOI:** 10.21956/openreseurope.23924.r68391

**Published:** 2026-02-17

**Authors:** Petteri Laihonen

**Affiliations:** 1 University of Jyväskylä, Jyväskylä, Finland

I read this article with great excitement, since it is a very helpful companion for researchers with experience in Citizen Science like me. The expansion of CS projects in the past ten years have brought about serious tensions around the definition of Citizen Science. Many researchers, me included, have noticed that especially in the academic world, there is hardly much agreement on either who is meant by "citizen" and what "science" entails nor what the relationship between the two can or should be. 

 The authors, curiously representing research institutions and including no "citizens", address many of the acute issues faced by researchers conducting CS projects. The answers the article brings forth are mostly laudably open, responsible, flexible and inclusive, perhaps bringing back those researchers to the field and ECSA that have already decided to use other term for their projects than CS.

 The main concern with the article is that it is framed as an empirical research article, whereas it reads more as a discussion note to already previously analyzed data. To address this, **I would suggest cutting the parts already presented elsewhere on the 10 Principles or the Characteristics, and focus on the new analysis and results. **That being said, the article certainly takes the discussion further and thus is a very needed encouraging contribution to the discussions of the "devilish" details of Citizen Science work globally. 

 Next, I will point to some specific issues for consideration to the authors:

 The abstract does not clearly indicate what is the novel contribution of the paper, e.g. stating that: "This paper introduces the *ECSA Characteristics of Citizen Science", *which refers to earlier work. Please add a sentence on the unique contribution of this paper in the abstract.

 The article opens with a historical narrative of CS, indicating a recent development, perhaps add a note on alternative histories, with a longer perspective, such as presented by Mahr et al. (2018). The introduction serves already now as a valuable state of the art source of most important literature. I would still suggest adding/updating some important (re)sources and taking a note on them: (refer to 4) is included, please take note on the newer version. 2) sources on cs in the arts and humanities are not represented equally in the literature review, event though in later parts they are often referred to as critically different from e.g. the natural sciences, which would warrant the better inclusion of such sources in the introduction, too.  Please add some sources in these fields if available to the authors. 3) two important recent developments in the ethics of CS could be included:(refer to 3) I also would find their inclusion in the parts referring to ethics helpful as they present the core state-of-the-art. 

 In the introduction I would reformulate the idea "to further professionalize the field", because it contradicts those historical accounts, where CS has been seen as an antidote to the (side effects of) professionalization of science, where the public or citizens are deliberatively excluded from the scientific research process (e.g. Mahr et al. 2018).

 With these very minor concerns in mind, which can easily be addressed with a minor revision, I welcome this publication and find it a very useful contribution for the many researchers tackling the issues in defining citizen science or interpreting the 10 principles in their fields and particular contexts.

If applicable, is the statistical analysis and its interpretation appropriate?

Not applicable

Is the study design appropriate and is the work technically sound?

Partly

Is the work clearly and accurately presented and does it engage with the current literature?

Partly

Are the conclusions drawn adequately supported by the results?

Yes

Are sufficient details of methods and analysis provided to allow replication by others?

Yes

Are all the source data and materials underlying the results available?

Yes

Reviewer Expertise:

Co-creative Citizen Science in Applied Language Studies, Participatory Research

I confirm that I have read this submission and believe that I have an appropriate level of expertise to confirm that it is of an acceptable scientific standard, however I have significant reservations, as outlined above.

## References

[1] Bernabe, R. de . la C., Carvalho, A. S., Dash, K. C. K., Koivisto, E., Kwamboka, L., Mezinska, S., Mbanya, V., & Strech, M. (2025). ROSiE General Guidelines on Responsible Open Science. Zenodo. https://doi.org/10.5281/zenodo.14957256.

[2] Mahr, D., Göbel, C., Irwin, A., and Vohland, K. 2018. “Watching or being watched: Enhancing productive discussion between the citizen sciences, the social sciences and the humanities”. In Hecker S., Haklay M., Bowser A., Makuch Z., Vogel J., and Bonn A. (Eds.), Citizen Science: Innovation in Open Science, Society and Policy (pp. 99–109). UCL Press. http://www.jstor.org/stable/j.ctv550cf2.14.

[3] ALLEA (2023) The European Code of Conduct for Research Integrity – Revised Edition 2023. Berlin. DOI 10.26356/ECOC.

[4] https://zenodo.org/records/14845396.

# Reviewer report: Reviewer response for version 2

**DOI:** 10.21956/openreseurope.23924.r68383

**Published:** 2026-01-24

**Authors:** Shane Orchard

**Affiliations:** 1 University of Canterbury, Christchurch, New Zealand

**Review of ‘Delineating the contours of citizen science: Development of the ECSA characteristics of citizen science”**

 Shane Orchard

 University of Canterbury | Te Whare Wānanga o Waitaha

**1. Introduction**

 This short comment responds to an invitation from Open Research Europe to provide a review of the paper “Delineating the contours of citizen science: Development of the ECSA characteristics of citizen science” (Version 2).

 The paper is clearly oriented around the objective of further developing the notion of ESCA Characteristics of citizen science which have been the focus of previous studies and initiatives (e.g., European Citizen Science Association (ECSA) 2015; Haklay et al., 2021). In the introduction, paragraphs 3–5 set out a clear rationale for further developing the existing ECSA characteristics literature to assist the needs of a broader range of stakeholders: particularly funders, policymakers and people new to citizen science. In contrast the original ECSA ‘10 Principles of Citizen Science’ was mainly designed to provide best practice guidance for existing citizen science practitioners (ESCA, 2015). Although the ESCA characteristics have already been published (Haklay et al., 2020a) along with a set of explanatory notes (Haklay et al., 2020b) and a scientific article that describes the methodology used for their development (Haklay et al., 2021), this paper makes a further contribution by examining the ESCA Characteristics in relation to the ECSA 10 Principles of Citizen Science and providing examples of their applications.

 The study has been generally well conceived and executed to address its stated objectives. Several amendments have also been made to Version 1 of the manuscript in response to previous review comments. I am generally supportive of the suggestions made by previous reviewers and the responses made by the authors are generally appropriate. These include streamlining of the Methods section to refer readers to existing publications or supplementary materials and the provision of additional information on the evolution of citizen science. Regarding the latter, however, much of the additional information is organized around the theme of a timeline (e.g., of evolving citizen science organisations and initiatives) whereas it may be better to instead focus on the specific contributions that have been made to the theme of defining the characteristics of citizen science. There are also a few other inconsistencies in the study’s framing and interpretation of results that could further improve the paper. These are briefly summarised in the following section.

**2. Specific comments**

*2.1 Abstract*

 Although the Abstract is otherwise sound, the first section (i.e., ‘Background’) does not clearly articulate the specific objectives for this paper in relation to previous publications on the ESCA Characteristics.

*2.2 Introduction*

 I have some concerns around the merits of statements made in the Introduction on the role of national associations which have reportedly ‘developed guidance to further professionalize the field’. Here in Aotearoa New Zealand (NZ) we prefer to use a framing around supporting and embracing the field in all its diverse forms and modalities. It can be argued that the goal of ‘professionalizing’ the field might actually be contrary to both the purposes and beneficial outcomes of some forms of citizen science, such as those based on local and traditional knowledge (e.g., see Jopling et al. (2024)). A further contradiction can be seen when comparing this material with the main conclusions of the paper and suggested benefits of adopting ‘an inclusive approach and language that allows the audience to define its own criteria depending on its needs, intended use and specific circumstances’. The main conclusions essentially present an argument around embracing diversity in the varied forms of scientific enquiries that might be considered to fall within the definition of ‘citizen science’ which requires that the diverse motivations and objectives of participants are fully recognised. I therefore suggest that the Introduction could be expanded to briefly traverse the existing literature that identifies and highlights this diversity (e.g., before moving on to describe the evolution of major organisations and initiatives in this field).

 Further to the above comment, the list of major national and international organisations that have arisen could be more clearly linked with initiatives and outputs that have either attempted to define the field, or offer insights for doing so (i.e., in the Introduction, paragraph 2). One option for doing this might be to wrap up this paragraph with a summary to that effect before moving on to summarise the work of scholars who have traced or commented on the development or scope of the citizen science field. This scholarly work offers a complementary perspective on the main theme of the Introduction and warrants a little more attention than currently provided (e.g., in a separate paragraph).

*2.3 Methods*

 The study design is generally appropriate. It is largely built around the results of previous work on the ESCA characteristics. Very few additional methods are used to develop this paper; they mainly consist of tabulated comparisons and the presentation of example applications. I support the steps already taken to streamline the Methods section of this paper. However, only the very last paragraph of the Methods section contains material that is specific to this paper.

*2.4 Results*

 The Results section presents a sufficient amount of new material to warrant this additional publication. However, I do not agree with the statement that suggests the Results provide a ‘novel contribution to the field by outlining the Characteristics themselves’. Such material is already found in the original ESCA Characteristics document and supporting explanatory notes (Haklay et al., 2020a; Haklay et al., 2020b). The first part of the Results section is essentially a summary of material already presented in the abovementioned documents (particularly Haklay et al., (2020b)) and it would perhaps be appropriate to state this directly.

 The second part of the Results section that presents a comparison of the ‘ECSA characteristics’ against the ECSA 10 Principles of Citizen Science is relatively brief but sound in its construction. The tabulated material (Table 1) is particularly helpful and includes an analysis of gaps or ambiguities in the ECSA 10 Principles which have been addressed in the ECSA Characteristics project.

*2.5 Discussion*

 The Discussion section provides a suitable summary of the paper but also a few inconsistencies that require further attention or explanation.

 These include:

 Paragraph 1 suggests that this paper explains how the statistical results from previous work have been transformed into explanatory outcomes, yet no statistical results are presented or even mentioned elsewhere in the paper. Therefore, this claim needs to be further explained or substantiated.

 Paragraph 3 describes an application of the ESCA Characteristics within a quality criteria framework that is used to screen resources for inclusion on the EU-Citizen.Science website. It would be good to provide further details of the quality criteria framework to assist the reader to identify the relevant connections (e.g., in Supplementary Materials). This could also provide an opportunity to further discuss how the ECSA Characteristics work has added value over-and-above the pre-existing ECSA 10 Principles of Citizen Science; consistent with the stated objectives for this paper.

 Paragraphs 7–10 of the Discussion focus on some of the current and potential applications of the ECSA Characteristics. Whilst this material is relevant it doesn’t specifically address the results of this study. However, paragraph 11 onwards rounds out the Discussion nicely by returning to the comparison between the ECSA 10 Principles of Citizen Science and ESCA Characteristics.

 The noted limitations of this Europe-centric work are appropriate given the substantial attention throughout the paper to potential applications within international frameworks, or to assist global-level comparisons or initiatives. The discussion of non-strict definitions and plurality of citizen science initiatives is particularly welcome, but no effort has been made to link these perspectives back to the existing literature that highlights their importance.

 The suggested opportunities around adaptation of the ECSA 10 Principles and Characteristics to other contexts and potential expansion to a global framework are also welcome and deserve further consideration. If proceeding towards a global framework of principles or characteristics along these lines it would be essential to adopt an inclusive approach with solid socio-cultural and geographical representation, particularly from the global south which is currently under-represented in many citizen science networks and initiatives.

*2.6 Conclusions*

 The structure of this section seems a little out of place since paragraph 3 is by far the most relevant to this study (e.g., it could be moved to become the opening paragraph). Additionally, the framing of paragraph 2 is not consistent with these aspects of the field because it suggests that social sciences, humanities, and grassroots co-creation of knowledge are ‘emerging approaches and disciplines’. All three disciplines actually pre-date the modern terminology around ‘citizen science’. It may be better, therefore, to reorient the Conclusions towards a summary of the new contributions provided by this paper (i.e., differences between the ECSA 10 Principles of Citizen Science and ESCA Characteristics).

**3. Summary**

**Is the work clearly and accurately presented and does it engage with the current literature?**

 The work is clearly presented but could better engage with the relevant literature in some sections.

 NB: a typo was noted in the Discussion section subtitle: The ECSA Characteristics in relation to the ECSA 10 Principles of Citizen Science

**Is the study design appropriate and is the work technically sound?**

 The study design is relatively brief since it mainly relies on existing work that has already been published, However, it is appropriate for the stated task and objectives.

**Are sufficient details of methods and analysis provided to allow replication by others?**

 Yes, all of the methods are clearly documented, particularly in supporting and supplementary materials.

**Are all the source data and materials underlying the results available?**

 Yes

**If applicable, is the statistical analysis and its interpretation appropriate?**

 Not applicable as there are no statistical analyses in the reported results (and none are needed).

**Are the conclusions drawn adequately supported by the results?**

 The conclusions are partly supported by the results but could be more clearly related to study objectives and its novel contributions. This also an opportunity to link the main conclusions back to existing literature that is consistent with them. There is also an interesting statement made in the Abstract that is not mentioned in the main text Results or Conclusions, as follows:

 ‘… it is beneficial to adopt an inclusive approach and language that allows the audience to define its own criteria depending on its needs, intended use and specific circumstances.’

 It would be great to incorporate this suggestion within the main text as appropriate

If applicable, is the statistical analysis and its interpretation appropriate?

Not applicable

Is the study design appropriate and is the work technically sound?

Partly

Is the work clearly and accurately presented and does it engage with the current literature?

Partly

Are the conclusions drawn adequately supported by the results?

Yes

Are sufficient details of methods and analysis provided to allow replication by others?

Yes

Are all the source data and materials underlying the results available?

Yes

Reviewer Expertise:

Socio-ecology, Participatory Science

I confirm that I have read this submission and believe that I have an appropriate level of expertise to confirm that it is of an acceptable scientific standard, however I have significant reservations, as outlined above.

## References

[1] : Postdigital Citizen Science and Humanities: A Theoretical Kaleidoscope. *Postdigital Science and Education* .2025;7(1) : 10.1007/s42438-024-00481-5 31-77 10.1007/s42438-024-00481-5

[2] : Contours of citizen science: a vignette study. *Royal Society Open Science* .2021;8(8) : 10.1098/rsos.202108 10.1098/rsos.202108 PMC838538534457323

[3] European Citizen Science Association (ECSA). (2015). Ten principles of citizen science. Berlin, ESCA. doi:10.17605/OSF.IO/XPR2N.

[4] Haklay, M., Fraisl, D., Greshake Tzovaras, B., Hecker, S., Gold, M., Hager, G., . . . Vohland, K. (2021). Contours of citizen science: a vignette study. Royal Society open science, 8(8). doi:10.1098/rsos.202108.10.1098/rsos.202108PMC838538534457323

[5] Haklay, M., Motion, A., Balázs, B., Kieslinger, B., Greshake Tzovaras, B., Nold, C., . . . Wehn, U. (2020a). ECSA's Characteristics of Citizen Science. Zenodo. doi:10.5281/zenodo.3758668.

# Response

**Published:** 2026-02-09

**Authors:** Dilek FRAISL

We thank the reviewer for the constructive and helpful feedback. Below, we summarize how each comment has been addressed.

 We revised the background section to more clearly articulate the specific objectives of this paper, as suggested. In response to comments on framing, we replaced the term “professionalize” with “strengthen support structures and legitimacy of the field in all its diverse forms and modalities”. We also added a new paragraph to the Introduction, following the opening paragraph, that engages with literature highlighting the diversity of citizen science practices, before moving on to the evolution of major organisations and initiatives.

 Additional text was included to explicitly link key national and international organisations and initiatives to the outputs and activities through which they have contributed to delineating and shaping the field. This appears immediately after the sentence beginning “UNESCO’s 2021 Recommendation on Open Science…”. We also added bridging text beginning with “Taken together, these organizational and global initiatives…” within the paragraph on domain-specific journals to better connect organizational developments with scholarly work and to give greater emphasis to the academic literature.

 To address the comment on the Methods section, we added an opening paragraph clearly outlining the methodology in two parts: elements building on previous work and the novel contribution of this study. This clarifies the additional methodological step taken here, specifically the identification of gaps and limitations in the ECSA 10 Principles and how the ECSA Characteristics can help address them.

 Regarding the Results section, we would like to clarify our intent concerning the  novel contribution of this paper. While the ECSA Characteristics were originally published on Zenodo to enable early and open access, this paper extends that work by situating the Characteristics within a peer-reviewed context and providing additional analysis and application.

 In the Discussion, we clarified that the statistical results underpinning the work are reported in Haklay et al. (2021) and explicitly noted that this paper builds on those findings. We expanded the description of the quality criteria framework used on the EU Citizen Science platform. We also added a paragraph addressing non-strict definitions and plurality and included an explicit statement in the final paragraph emphasizing the need for inclusive, globally representative approaches, particularly involving the Global South.

 Finally, the Conclusions were reorganized by moving the former third paragraph to the opening of the section and refocusing the discussion on the core contribution of the paper. References to social sciences, humanities, and grassroots knowledge as “emerging disciplines” were removed, recognizing them instead as established approaches. The section was also tightened to reinforce the overall contribution and significance of the paper.

# Reviewer report: Reviewer response for version 1

**DOI:** 10.21956/openreseurope.21006.r60197

**Published:** 2025-11-05

**Authors:** Isabelle Bonhoure

**Affiliations:** 1 University of Barcelona, Barcelona, Catalonia, Spain

The paper “Delineating the contours of citizen science: Development of the ECSA characteristics of citizen science” offers valuable insights by revisiting the ECSA 10 Principles (2015) considering the more recent ECSA Characteristics (2020). 

 The presentation of scientific work is clear and accurate, and the paper draws upon a wide range of relevant references. While the literature on definitions and terminology in citizen science (CS) is extensively reviewed, limited information is provided regarding the evolution of CS between the formulation of the ECSA 10 Principles (2015) and the present (2025). As the authors note in their conclusions, CS is a rapidly growing and dynamic field. Since 2015, it has experienced a substantial increase in the number of scientific publications and a notable diversification of research topics. Including a brief overview of this evolution—based on review papers and/or systematic literature reviews—would help readers better contextualize the current study. 

 The study design is sound and appropriate, and the paper brings valuable academic insights. Although the work is already solid and meaningful, its relevance could be further enhanced if the authors situated their contribution within the broader timeline of CS. When the ECSA 10 Principles were developed in 2015, CS was still a relatively new approach—at least within the European context. By the time the ECSA Characteristics were formulated in 2020, the field had already evolved, and by 2025, CS has reached a more mature stage. Making this evolution explicit within the paper would help readers better understand the context of the field and clarify the gaps the authors identify. 

 Furthermore, the vignette cited serves as a clear example of an activity that most survey respondents are not considering to be CS. While its analysis yields interesting insights, it could also be valuable to include an example that is clearly recognized as CS—or even a more controversial vignette where respondents expressed divergent views—to further discuss the inclusion and boundaries of certain practices within the CS framework. 

 The Methods section provides sufficient detail. However, since part of the information has already been published elsewhere (Haklay et al., 2021), certain parts—particularly those describing the vignette survey—could be summarized to avoid redundancy. 

 The source data, including formerly published articles and Zenodo reports, are fully documented and available.  

 The Conclusions are well supported by the results presented in the paper. As the authors note, CS is a rapidly evolving field. It could strengthen the paper if the authors elaborated in the conclusions on how their results both reflect this evolution and offer a framework flexible enough to accommodate future developments in CS, while remaining grounded in its core principles.

If applicable, is the statistical analysis and its interpretation appropriate?

Not applicable

Is the study design appropriate and is the work technically sound?

Partly

Is the work clearly and accurately presented and does it engage with the current literature?

Yes

Are the conclusions drawn adequately supported by the results?

Yes

Are sufficient details of methods and analysis provided to allow replication by others?

Yes

Are all the source data and materials underlying the results available?

Yes

Reviewer Expertise:

Citizen Science, Citizen Social Science

I confirm that I have read this submission and believe that I have an appropriate level of expertise to confirm that it is of an acceptable scientific standard, however I have significant reservations, as outlined above.

# Response

**Published:** 2025-12-02

**Authors:** Dilek FRAISL

We thank the reviewer for their positive and constructive feedback.

- To address their comments, we have expanded the introduction to describe the evolution of CS from the publication of the ECSA 10 Principles in 2015 to the present.
- We also draw attention to relevant (systematic) review papers that examine this evolution from various thematic perspectives. In addition, we have positioned our contribution within this broader developmental trajectory and clarified its continued relevance to current CS practice, including how it addresses the gaps identified.
- Regarding the vignette-related feedback, we agree that this material is both interesting and pertinent. We have therefore added a clarification and a link to the relevant publication (Haklay et al., 2021). As the examples and discussion requested by the reviewer are already detailed in that work, repeating them here would create unnecessary duplication. We chose this approach to remain consistent with the reviewer’s following observation that, because parts of the material have been published previously (Haklay et al., 2021), sections, particularly those describing the vignette survey, should be summarised to avoid redundancy. We believe this strategy helps keeps clarity and focus in the current paper.
- Finally, in the methods section, we have streamlined the content, especially the description of the vignette survey, and now refer readers to Haklay et al. (2021) for full details.
- In the conclusions, we have added text reflecting on the evolution of CS and mentioning how future developments can be incorporated within our proposed framework.

# Reviewer report: Reviewer response for version 1

**DOI:** 10.21956/openreseurope.21006.r60199

**Published:** 2025-09-29

**Authors:** Dolores Mumelaš

**Affiliations:** 1 National and University Library in Zagreb, Zagreb, Croatia

This research article is written with clarity and coherence, providing a logical continuation of the previous study on the ECSA characteristics of citizen science. The text and its findings are supported by a solid body of relevant literature from experts in the field.

 A particular strength of this work lies in its critical examination of the ECSA Principles. The study identifies existing ambiguities and gaps, and it demonstrates in a concrete manner how the Characteristics can serve as a framework to address these challenges.

 The study design is appropriate, and the work is technically rigorous. The methodology is clearly described, easy to follow, and well suited for this type of research, allowing replication. The article is further enhanced by numerous well-constructed figures that effectively illustrate the results and facilitate comprehension. In addition, all underlying data and materials are made available, which reinforces the transparency and reliability of the work.

 The conclusions are clear, insightful, and valuable for advancing the field of citizen science. This is a robust and meaningful contribution to the theoretical development of the discipline. It also provides a strong foundation for future research on this topic and has the potential to stimulate further investigations in related areas.

If applicable, is the statistical analysis and its interpretation appropriate?

Not applicable

Is the study design appropriate and is the work technically sound?

Yes

Is the work clearly and accurately presented and does it engage with the current literature?

Yes

Are the conclusions drawn adequately supported by the results?

Yes

Are sufficient details of methods and analysis provided to allow replication by others?

Yes

Are all the source data and materials underlying the results available?

Yes

Reviewer Expertise:

Citizen science

I confirm that I have read this submission and believe that I have an appropriate level of expertise to confirm that it is of an acceptable scientific standard.

# Response

**Published:** 2025-12-02

**Authors:** Dilek FRAISL

We thank the reviewer for this helpful feedback and positive decision.
